# Cold ablation robot‐guided laser osteotomy in hand, wrist and forearm surgery—A feasibility study

**DOI:** 10.1002/rcs.2438

**Published:** 2022-07-08

**Authors:** Philipp Honigmann, Maximilian Hofer, Sibylle Hirsch, Marta Morawska, Magdalena Müller‐Gerbl, Florian M. Thieringer, Enrico Coppo

**Affiliations:** ^1^ Hand and Peripheral Nerve Surgery Department of Orthopaedic Surgery and Traumatology Kantonsspital Baselland (Bruderholz, Liestal, Laufen) Bruderholz Switzerland; ^2^ Department of Biomedical Engineering Medical Additive Manufacturing Research Group (MAM) University of Basel Allschwil Switzerland; ^3^ Department of Biomedical Engineering and Physics Amsterdam UMC University of Amsterdam Amsterdam Movement Sciences Amsterdam The Netherlands; ^4^ Faculty of Medicine University of Basel Basel Switzerland; ^5^ Advanced Osteotomy Tools AG Basel Switzerland; ^6^ Institute of Anatomy University Basel Basel Switzerland; ^7^ Department of Oral and Cranio‐Maxillofacial Surgery University Hospital Basel Basel Switzerland

**Keywords:** cold ablation laser osteotomy, forearm, hand, laser, navigation, osteotomy, wrist

## Abstract

**Introduction:**

Traditional bone surgery using saws and chisels is associated with direct contact of instruments with the bone causing friction, heat and pressure and hence, damaging the bone and the surrounding soft tissues.

**Method:**

Cold ablation laser osteotomy offers new possibilities to perform corrective osteotomies in the field of bone surgery. We introduce the technology of navigated cold ablation robot‐guided laser osteotomy, present potential applications, and preliminary pre‐clinical cadaver test results in the field of hand‐, wrist‐ and forearm surgery.

**Results:**

The cadaver tests showed first promising results for corrections in all planes and axes using different cutting patterns.

**Conclusion:**

Cold ablation laser osteotomy seems to be a feasible new method to perform osteotomies in the field of hand‐, wrist‐ and forearm surgery. Primary osseous stability could be achieved using various cutting patterns which could lead to reduction of the amount of hardware required for osteosynthesis. Further tests are required to proof the latter and precision.

## 1 INTRODUCTION

Bone surgery is often associated with the use of rather crude instruments.^1^ The size of the tools often increases with the size of the bones, the larger the bone—the larger the instruments. Electrically or air‐powered, oscillating saws are still the widespread tools used to cut bones. Drills and chisels can also be used to perform osteotomies. All these instruments need a direct contact with the bone, causing friction, heat and pressure and hence, damaging the bone and the surrounding soft tissues.^2^, ^3^ Especially sawblades can create an amorphous and mineral enriched carbon layer during their contact with the bone, which can hamper bone regeneration and in the worst case, lead to bone necrosis.^4^

Developed by Shafer in 1958, piezoelectric devices, which induce ultrasound frequencies on the surface have been introduced into bone surgery and are especially used in cranio‐maxillofacial surgery.^1^, ^5^ Compared to oscillating saws and drills, piezoelectric devices cause less damage to the bone and soft tissue, enable faster bone healing and higher precision while cutting.^6^, ^7^, ^8^

All the mentioned cutting tools have a limitation of physical cuts and geometries due to their blade size and manual guidance. Even in case of robot‐guided surgery, there is still a contact between the instrument and the bone.

In the nineties, lasers were introduced and modified for thermal bone ablation. Especially an erbium‐doped yttrium aluminium garnet (Er:YAG) laser with the wave length of 2943 nm was superior to drill‐osteotomies in terms of bone healing.^9^ Recent pre‐clinical studies showed that this contact‐ and vibration‐free technology allows to perform osteotomies with high precision, smooth cutting surfaces and less damage to the surrounding soft tissues.^3^, ^4^, ^10^ The osteotomy planes showed no smear and no carbonisation, leaving the bone channels open and preserving the trabecular architecture, which facilitates the passage of cells into the osteotomy site for rapid onset of bone healing. Several pre‐clinical and clinical studies have shown that the healing outcome when using an Er:YAG laser with water cooling is comparable to that of conventional mechanical osteotomy and piezoelectric surgery.^4^, ^11^, ^12^, ^13^, ^14^, ^15^, ^16^

Cutting patterns, such as sinus‐, puzzle‐, tri‐ and rectangular, straight and spiral shaped are possible to increase bone contact surface and facilitate achievement of final position of the fragments. Correction of deformities like malunions in all planes and rotational axes (x, y, z) are possible.

Results of a first‐in‐man clinical study of cold robot‐guided navigated laser midface‐osteotomies in 14 consecutive patients, who required orthognathic surgery showed promising results.^17^

So far, no applications of this promising technology in hand‐, wrist‐ and forearm surgery has been described in the literature. We introduce the technology of navigated cold ablation robot‐guided laser osteotomy, present potential applications, and preliminary pre‐clinical test results in the field of hand‐, wrist‐ and forearm surgery.

## 2 TECHNOLOGY

**FIGURE 1:**
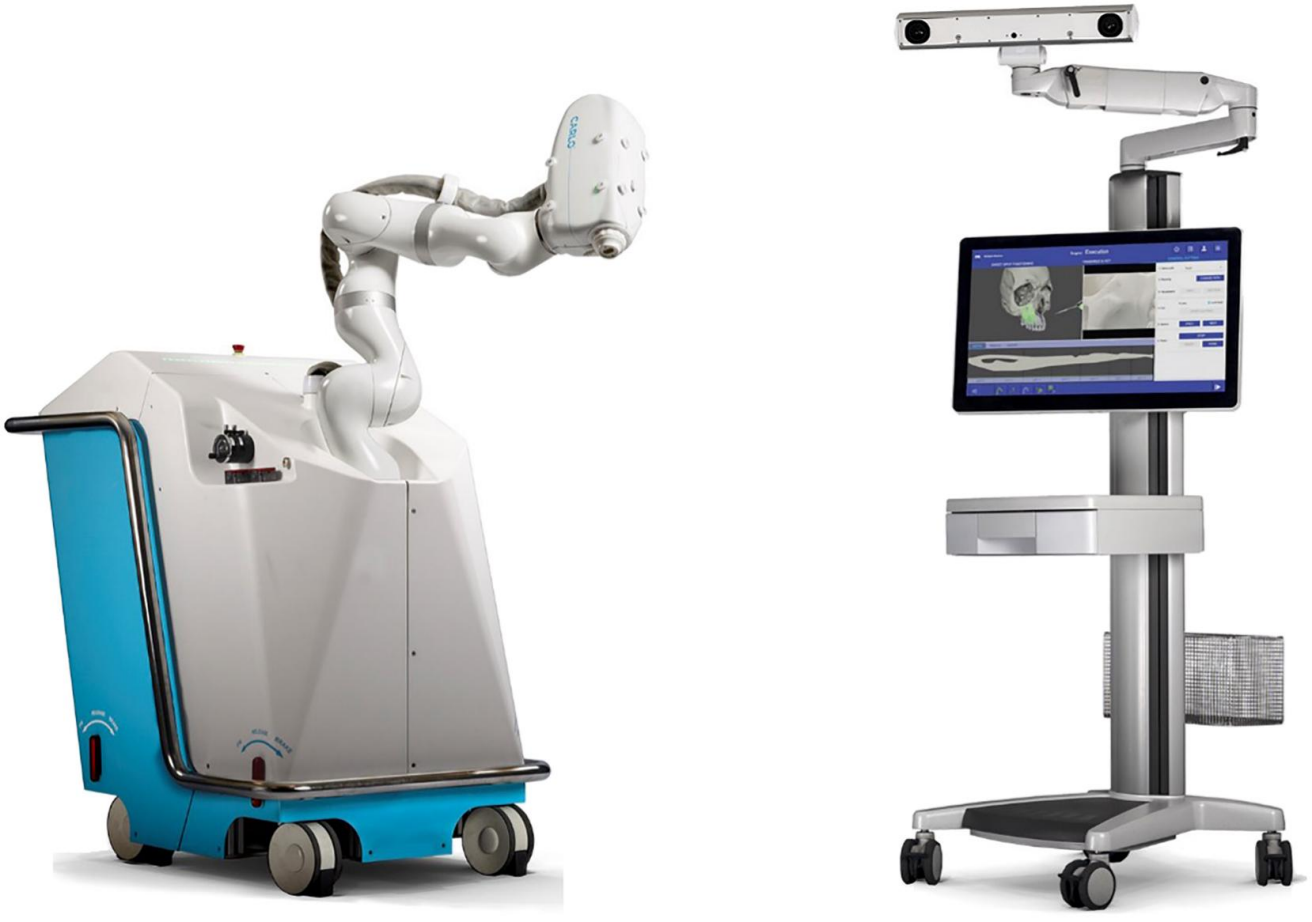
The Cold Ablation Robot‐guided Laser Osteotome (CARLO primo) and the optoelectric tracing system

Cold ablation robot‐guided laser osteotome (CARLO^®^) is a miniaturised ablation laser with an optical system in a compact casing and mounted on a tactile surgical robot (KUKA Light Weight medical grade Robot, Augsburg, Germany), which is controlled by a navigation system (Figure 1). The system uses Yttrium Aluminium Garnet (YAG) doped with Erbium (Er) (Er:YAG), with a wavelength of 2943 nm, which corresponds to peak absorption coefficient for water and hydroxyapatite (a major component of bone). During the cold ablation process, the energy of laser pulse hitting the bone tissue heats up the water content of the bone and vaporizes it. The increase in local pressure causes ‘micro‐explosions’, breaking up the bone structure. The debris is being expelled immediately and at high velocity, providing a clean‐cut line with preservation of the callous structure of the bone.^14^ Due to the speed of expulsion, as well as sweeping motion of the laser head during ablation, minimal thermal energy reaches the osteotomy site, resulting in a thermal profile comparable to mechanical instruments.^18^ What is more, potential thermal damage to the surrounding tissues can be practically eliminated by spraying water into the surgical field.^2^ There is also a sort of pooping sound during the ablation process which is a photoacoustic effect which is due to a shock wave induced by fast distribution of the laser energy^19^ (Video 1).

CARLO^®^ also has an Optical Coherence Tomography (OCT)‐ based depth control system to visualise the current cutting level and avoid soft‐tissue damage.

The safety and efficacy of CARLO^®^ was confirmed in 2019 during first‐in‐man clinical study (ClinicalTrials.gov Identifier: NCT03901209), without intraoperative complications or technical failure and was granted CE‐1250 certification in 2021.^17^, ^20^

**FIGURE 2:**
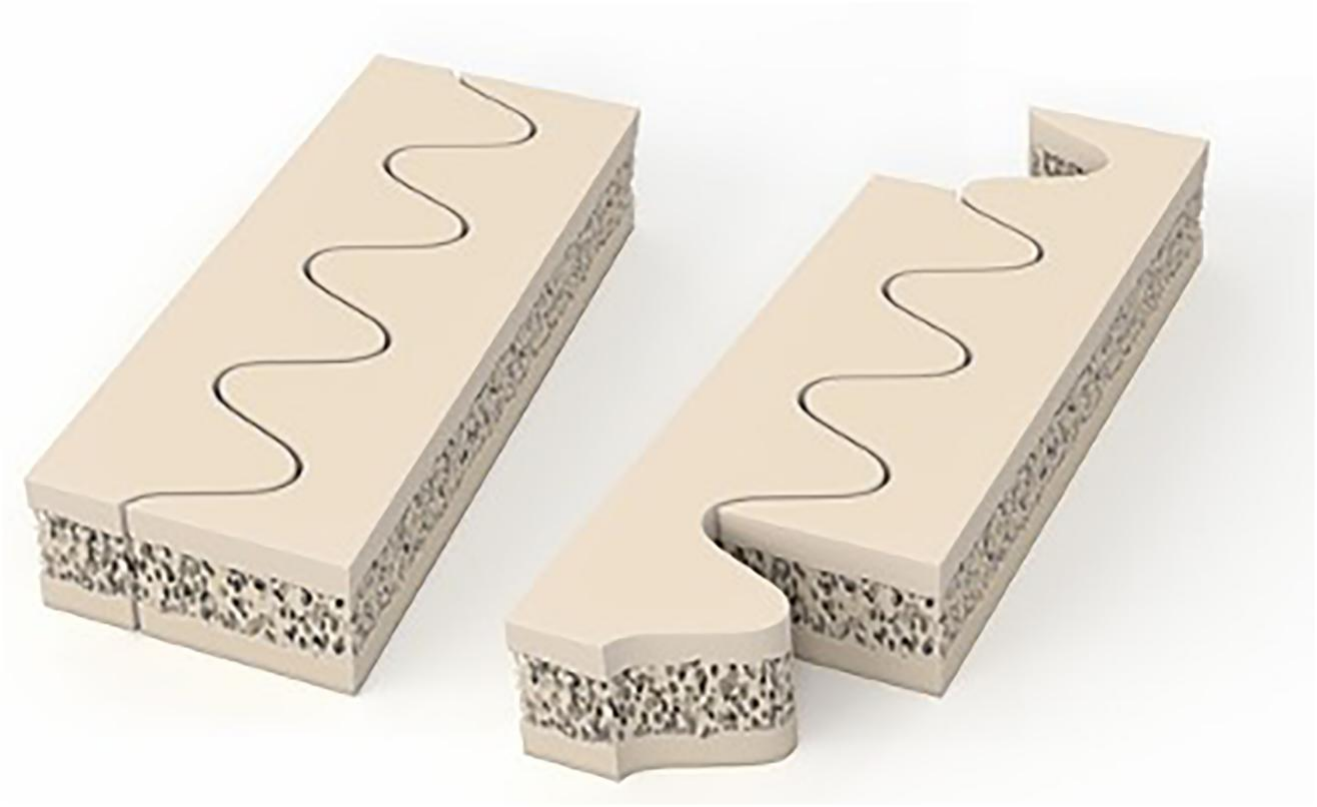
Graphical presentation of a sine‐wave cut and translation (with kind permission of AOT)

The navigated and controlled free movement of the robotic arm allows for changing of directions during the osteotomy process. This freedom reveals new possibilities for combined cutting patterns which are impossible to precisely cut in manually fashion. The frequently used pattern is the sinae shaped cut (Figure 2), which avoids weak bone spikes and fragile bony parts. Many other shapes are possible and can be combined depending on the required correction of planes and axes (Table 1). For translational correction in one plane, a step‐cut alike pattern is feasible to correct for length. A semi‐circular pattern like in dome osteotomies can be used for rotational corrections. Both, a sinae‐ or step‐cut pattern in a semi‐circular pattern in a frontal, sagittal or coronal plane can be used for the correction of length and angle. A torsion of the plane of the cut pattern from proximal to distal could be used to correct for length and axial rotation.

**TABLE 1:** Feasible cutting pattern

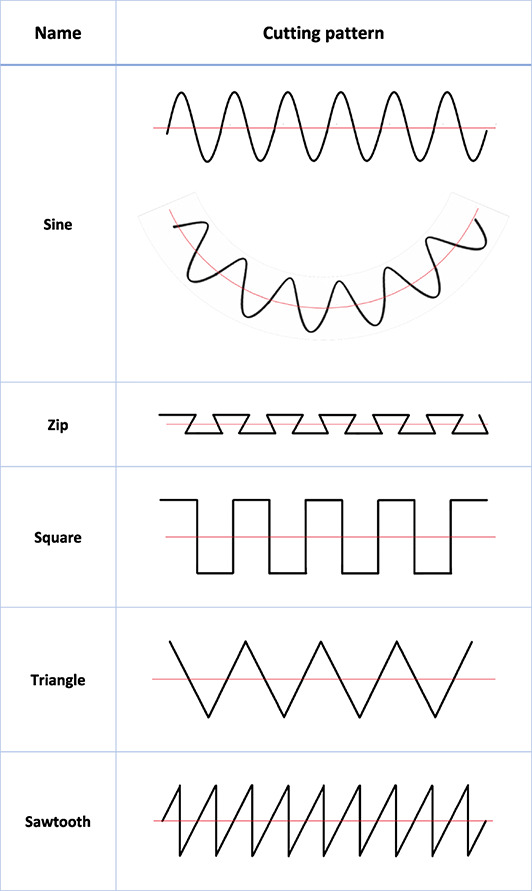

### 2.1 Potential hazards to patient and staff

The OCT allows for depth control and prevention of soft tissue damage while cutting and additionally, the soft tissue and other vulnerable structures can be protected by using a thin spatula/plate placed directly under the bone in the path of the laser. In the future, the OCT will use a laser to control the depth. The laser head immediately stops when the head is touched or moved from the outside to prevent uncontrolled cutting. The staff needs to wear eye protection as this laser does potential harm to the retina. This protection is always used while operating a laser instrument.

The staff should be aware not to interfere with the laser beam while operating as it does also damage the skin of the surgeons and scrub nurses' hand.

## 3 SURGICAL APPLICATIONS

Typical applications for the cold ablation robot‐guided laser osteotome are mal‐ and nonunions in the hand, wrist and forearm. Also, bone‐grafting procedures with complex patterns are possible to achieve high precision of the reconstruction and the best fit of the graft.^21^

Another application can be ablation procedures, such as bone debridement in cases of osteomyelitis to enhance fast ingrowth of healthy cells into the affected area.^22^

### 3.1 Osteotomies of the distal forearm bones

The most common fracture of the human body is the distal radius fracture.^23^, ^24^, ^25^ Irrespectively from the treatment, 11%–23% of all patients develop a symptomatic malunion. A frequently performed procedure is therefore the corrective osteotomy of the distal radius due to symptomatic malunion of a distal radius fracture.^26^, ^27^ Computer assisted analysis and planning of the osteotomy has been established as the gold standard and to facilitate the surgical procedure, additive manufactured guides are frequently used.^28^, ^29^, ^30^, ^31^, ^32^, ^33^

**FIGURE 3:**
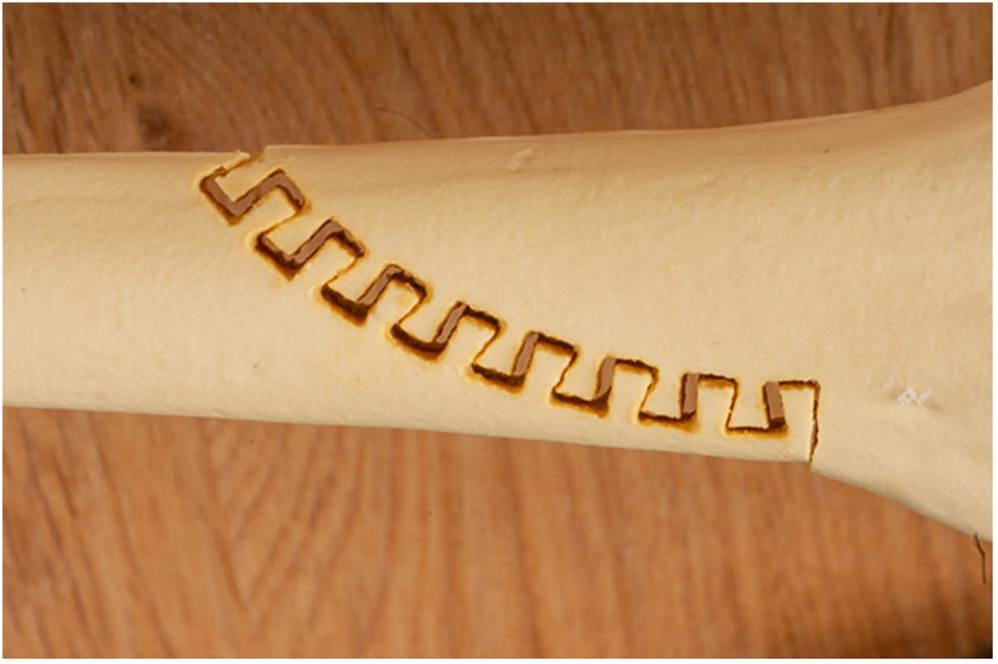
Rectangular shaped cut of the metaphyseal radius to correct for length and radial inclination

The planning process of the osteotomy using CARLO^®^ requires also a digital workflow with the assessment of the malunion and planning of the surgical intervention. In contrast to the conventional method, no intraoperative patient specific instrumentation is required. Figure 3 shows an example of a rectangular shaped cut of the distal metaphyseal radius done by CARLO to correct for radial inclination and length in a Synbone model (Synbone AG, Zizers, Switzerland).

**FIGURE 4:**
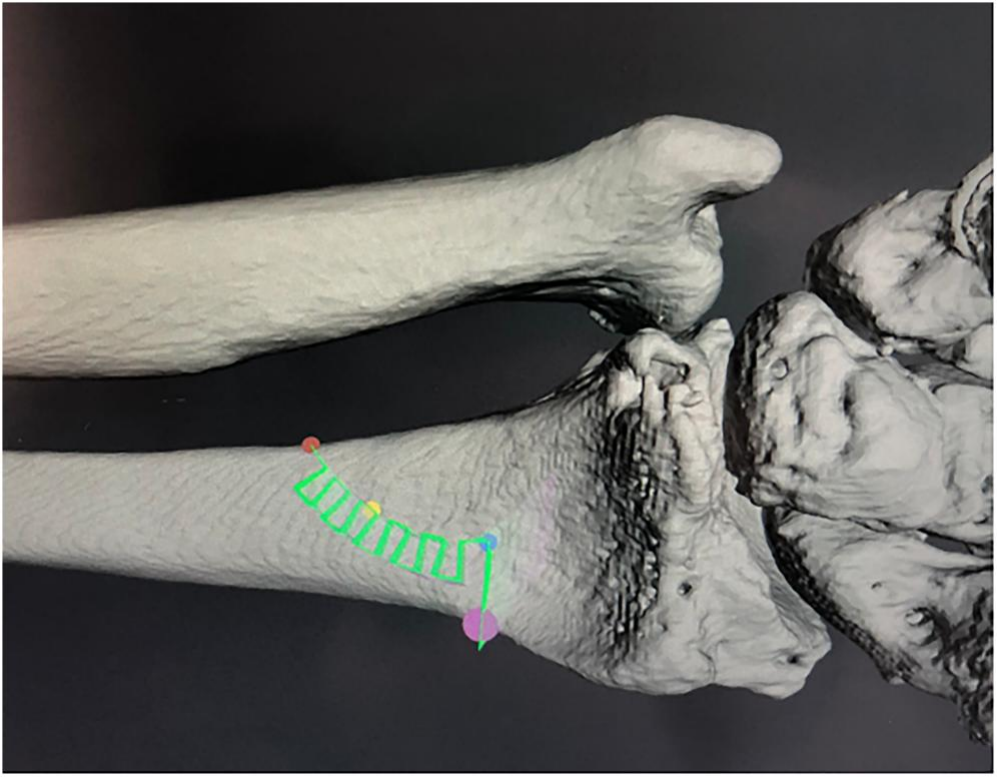
Computer assisted planning of the cut after surface registration

**FIGURE 5:**
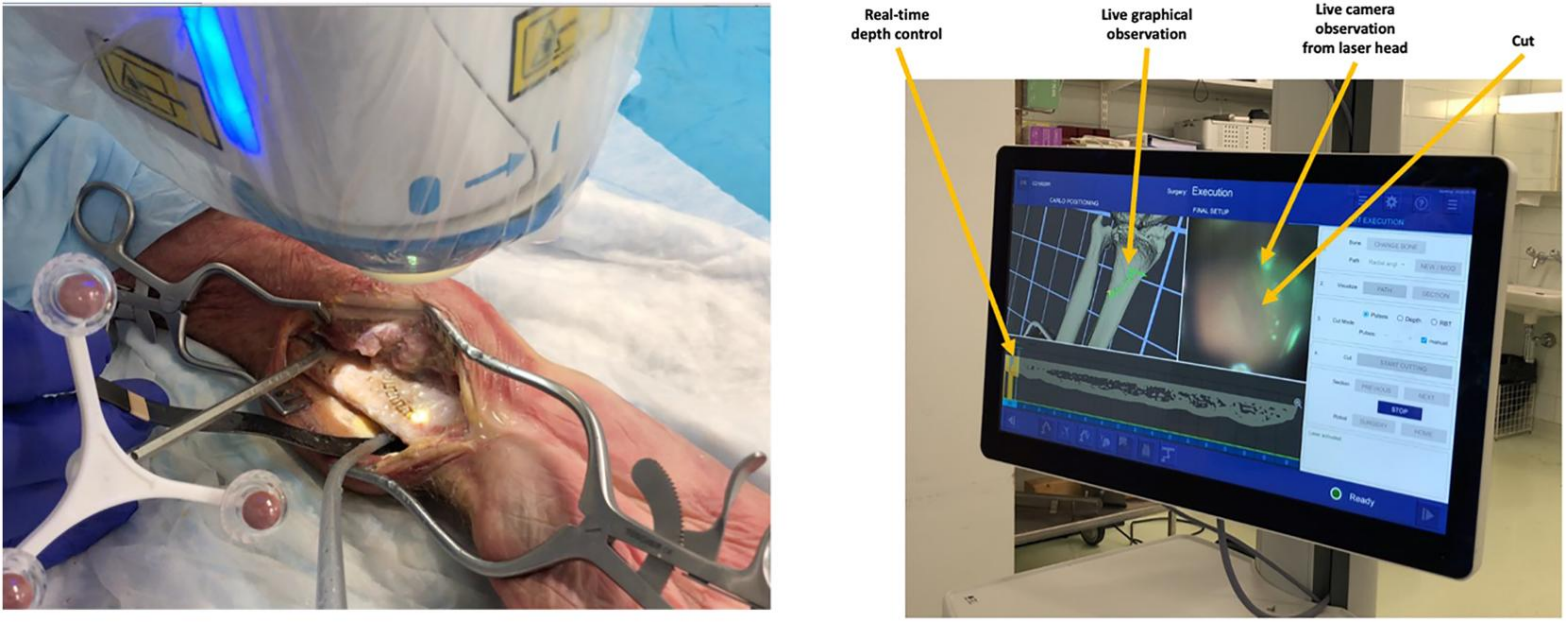
Performing the osteotomy (left) and real‐time observation of the navigation (right)

**FIGURE 6:**
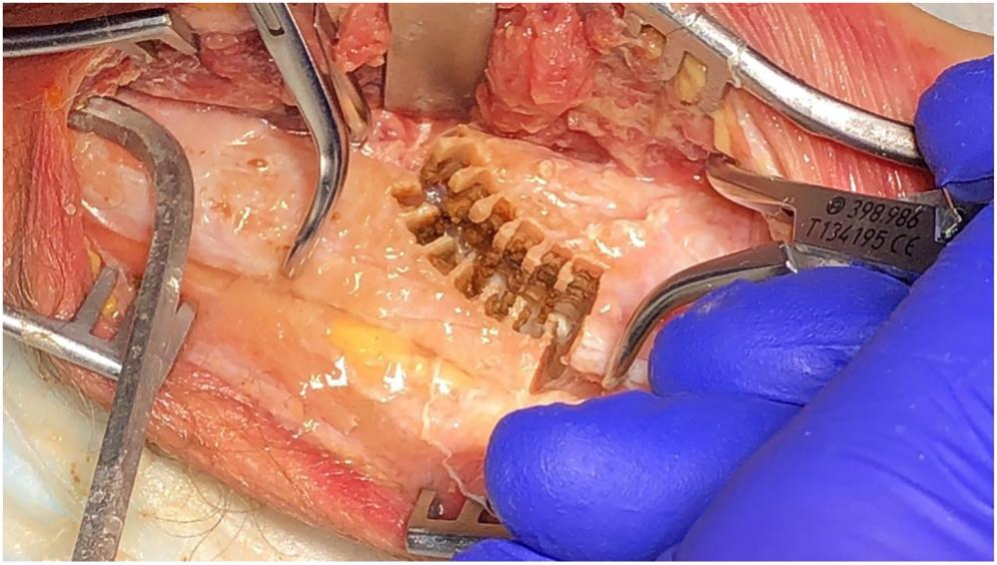
Final result after completion of the cut without carbonisation

Frequency, amplitude, and radius of the cut determine the amount of correction, which can be simulated before the intervention to assure for a precise result (Figure 4). The whole procedure is real‐time controlled by the navigation system of CARLO (Figure 5, Video 2) and takes about 10–15 Mins. The result after the procedure in a human cadaver shows a precise cut without any signs of carbonisation (Figure 6).

Any other type of single‐ or multi‐level diaphyseal correction is realistic and complex reconstructions using a vascularised fibula bone graft would be possible with the already published results of reconstruction of the lower jaw.^21^, ^34^

Another frequently performed procedure is the ulna shortening in cases of ulno‐carpal impaction. Typically, three osteotomy techniques are performed: transverse, oblique and step‐cut.^35^, ^36^, ^37^, ^38^, ^39^, ^40^, ^41^ Transverse and oblique osteotomies are facilitated using parallel saw blades and a fixation system.^42^, ^43^, ^44^ Due to the final fixation onto the plate as a reference, rotational and translational errors are possible using these devices.

**FIGURE 7:**
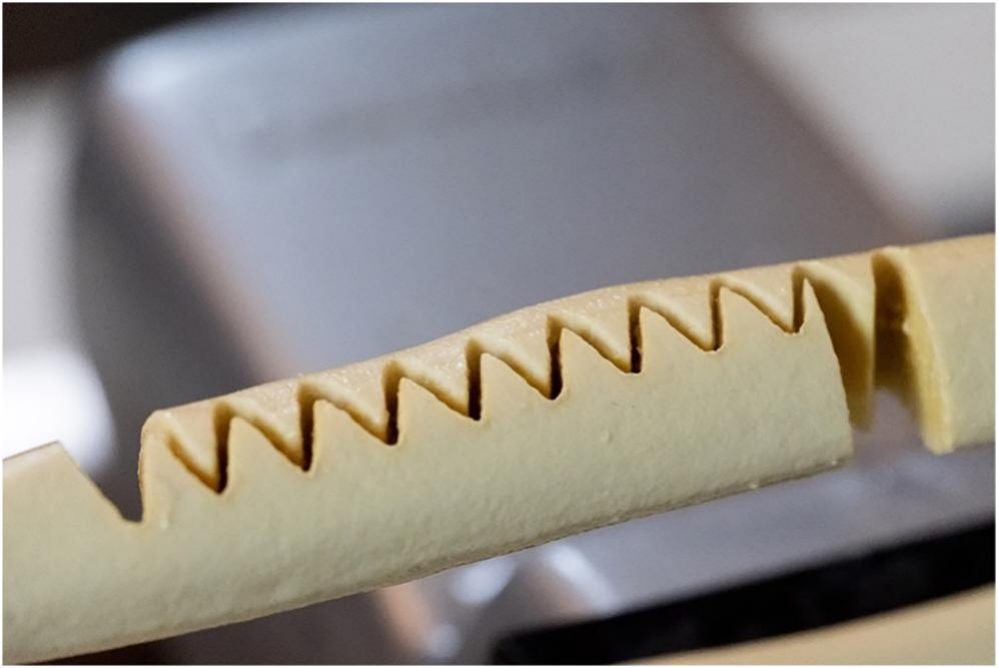
Sawtooth pattern for ulna‐shortening

**FIGURE 8:**
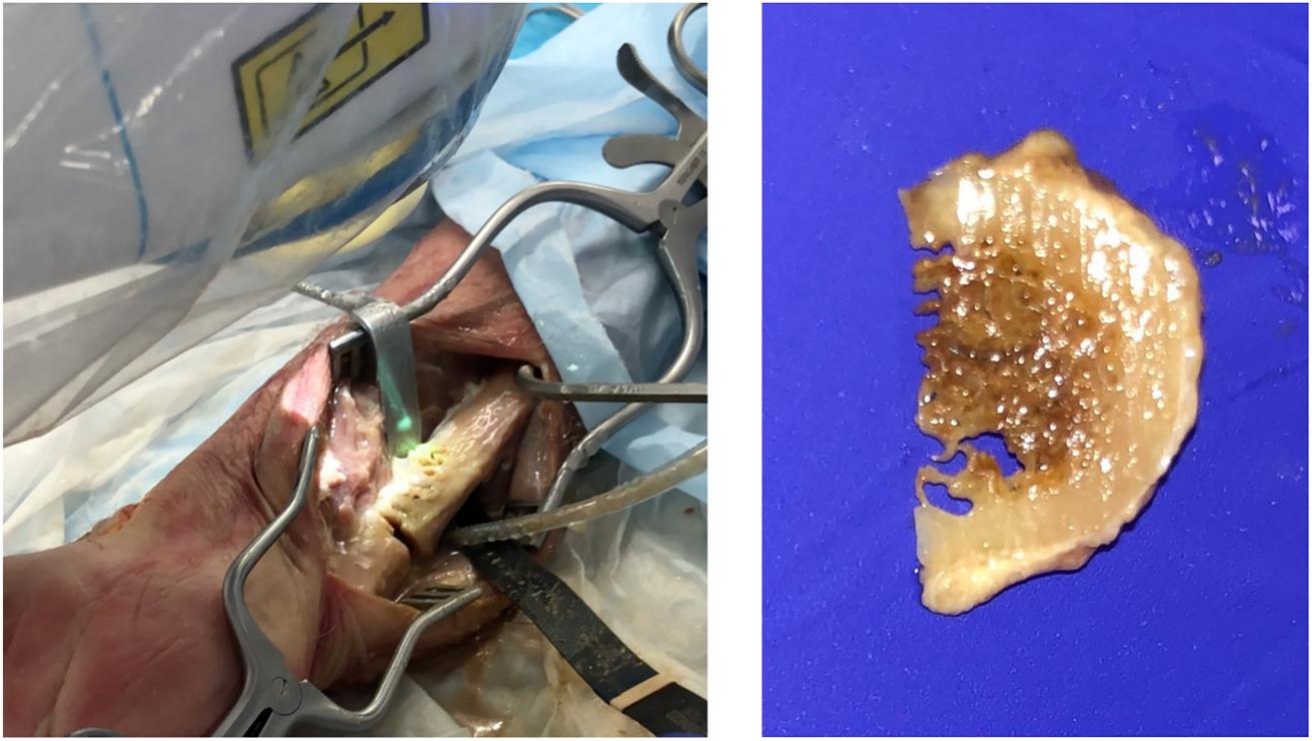
Ulna‐shortening osteotomy (left); resected distal bone part (right)

**FIGURE 9:**
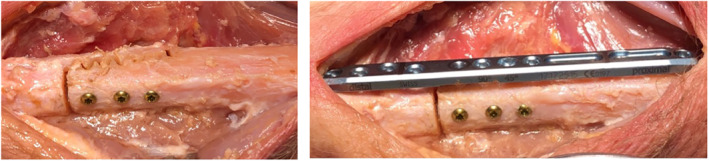
Final situation with 3 lag screws (left) and compared to conventional fixation plate (right)

A primary stable pattern for ulna‐shortening using CARLO^®^ is displayed in Figure 7. Due to the sawtooth design of the cut, a precise primary stability and best fit are achieved. Equal to the step‐cut linear osteotomy, the bone is shortened proximally and distally to attain the required amount of shortening and the pattern is adapted to achieve the best final fit. The cadaver test shows a symmetric and non‐carbonised bone cut, also on the resected bone piece (Figure 8). Absolute stability was achieved using 3 lag screws size 2.5 mm, perpendicular to the osteotomy. In contrast, a standard bulky plate, which is about twice as long as the construct and has the disadvantage of possible disturbance is shown (Figure 9).

**FIGURE 10:**
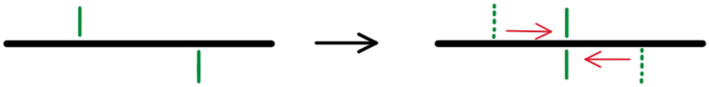
Straight cut with markings or pre‐shot screwholes (green lines) for standard plate as an orientation for the correction

In a second stage, the drill holes for the screws could be pre‐shot with CARLO, ablating the channel until the required diameter is achieved like sketched in Figure 10.

### 3.2 Osteotomies of the metacarpal and finger bones

**FIGURE 11:**
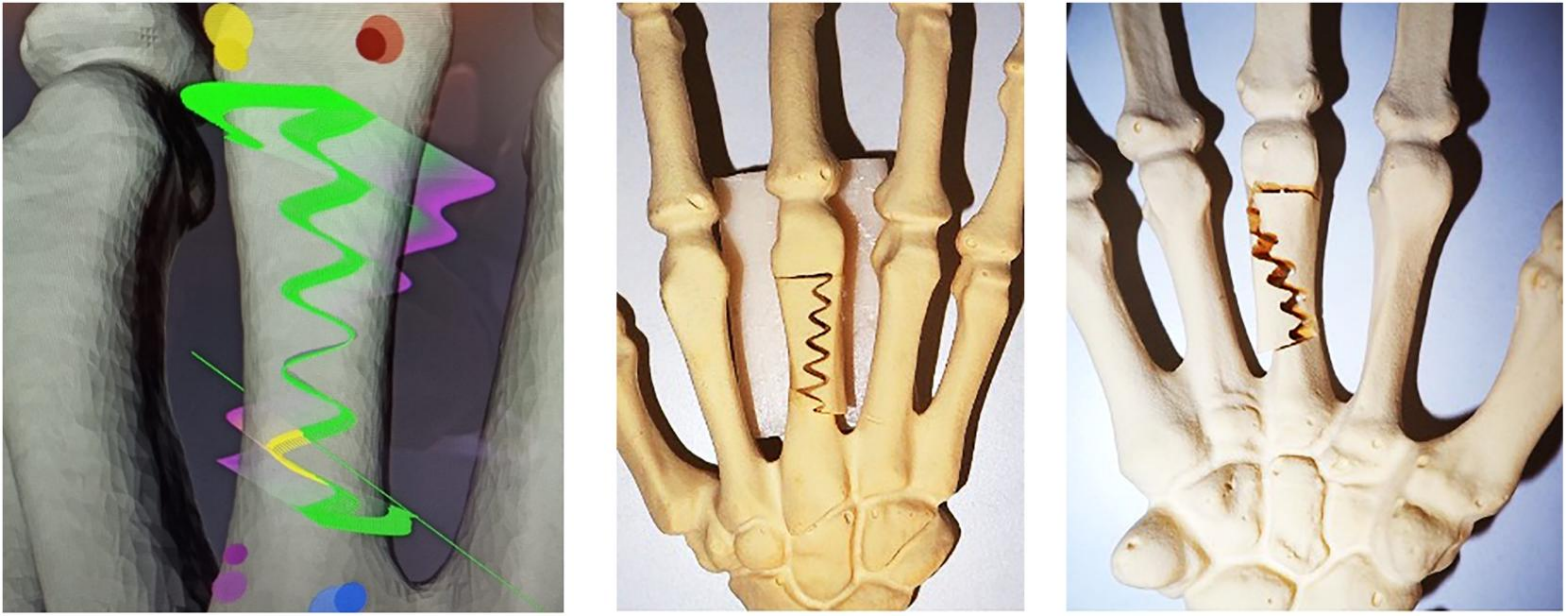
Planning (left) and final result of the osteotomy (middle: dorsal view; right: palmar view)

We simulated a complex osteotomy for the correction of rotation and length in a metacarpal malunion. The osteotomy was carried out in a Synbone model in a first step to assess feasibility. The sinae cut had a rotational component and was carried out without any problems (Figure 11). We realized the bone bridge proximally, however distally of the cut the bone model was weak with the potential of breakage. In the cadaver, we therefore changed the pattern according to a sine‐cut pattern with a rotational component, leaving a sufficient bone bridge at the start and end of the osteotomy.

Osteotomies of the proximal and intermediate phalanx of the finger using oscillating saws or chisels have the risk of damaging the flexor tendons. With the laser osteotome, this risk could be minimised with depth control (OCT).

## 4 FUTURE PERSPECTIVES

With the precision and freedom to correct for multiplanar and rotational axes, we see many more applications of CARLO^®^ in the field of hand, wrist and forarm surgery. Carpal surgery, especially scaphoid nonunion surgery could be facilitated and the osteotomies could be less damaging to this delicate bone. First case series of volar and dorsal navigated, and also robotic‐assisted placement of screws in scaphoid fractures are promising.^45^, ^46^, ^47^, ^48^, ^49^ These results, especially in terms of precision and operating expenses, should be considered for the treatment of scaphoid nonunions when using CARLO^®^.

The harvest of bonegrafts for the reconstruction might be possible in the region of the distal radius or olecranon. For iliac bone grafts, a CT‐scan would be necessary and it remains questionnable whether higher precision of the size of the bonegraft will be achieved.

One joint‐preserving treatment of early stages of osteoarthritis of the thumb carpo‐metacarpal joint is an extension osteotomy of the base of the metacarpal I by resection of a wedge and wire or plate osteosynthesis.^50^, ^51^, ^52^ CARLO^®^ could perform the closing wedge osteotomy and predetermine the final plate position by preshooting the screw holes.

The technique of pre‐marking the plate position and/or pre‐shot of screw holes could also be used for fracture reposition and fixation for example, in distal radius fractures, which are mostly assessed with CT‐scans that can be used for pre‐surgical planning of the laser intervention.

## 5 DISCUSSION

We introduced the technology and presented the potential applications and benefits of cold ablation robot‐guided laser osteotomy in the field of hand, wrist and forearm surgery.

There are several benefits of the use of CARLO^®^ in this field. The main benefits of CARLO^®^ are the ability to perform contact‐ and debris‐free osteotomies according to a preoperative virtual planning without the need for cutting guides and independent of surgeon's manual skills. The pre‐surgical plan can be uploaded directly to the device, which can execute the cuts contact‐free, with high precision and the possibility to be adapted intraoperatively. The osteotomy procedure itself, including planning and set‐up, is time consuming but compared with the whole process in terms of material/printer costs and manpower of pre‐operative design and the manufacturing process of additive manufactured guides, it seems to be comparable in the end. Future indication‐focussed feasibility studies should concentrate on the time and cost‐benefit aspect as well.

The ability to use various cut geometries, as shown in Table 1, offers a new possibility of precise change of bone length and angular correction, with better bone‐to‐bone contact and higher primary stability.^21^ The use of bioresorbable screws and/or allograft bone pegs could avoid any secondary intervention for hardware removal.

The combination of high precision, geometric cuts and timesaving during presurgical planning, in comparison to conventional state‐of‐the‐art techniques, could prove highly beneficial to the patients. Finally, as mentioned above, CARLO^®^ osteotomy could result in faster bone healing, compared to conventional methods, which need to be proved in future clinical studies.

The CARLO^®^ device relies on registration, which matches the virtual model with patients' anatomy using established landmarks. The point‐to‐point registration of the CARLO^®^ navigation has been proven and validated in several clinical studies in Cranio‐Maxillofacial Surgery, with average registration error of 0–1.9 mm (mean 0.8 mm).^13^, ^17^, ^21^ In case of radial or ulnar bone, there are no obvious anatomical landmarks that can be used for registration. Therefore, in addition to point‐to‐point registration, ICP (Iterative Closest Points) cloud can be used to match surface of the bone with the virtual model.

We are aware of the limitations of this first report of the use of CARLO^®^ in smaller bones. The results of in vitro and cadaveric tests need to be transferred and validated in patients during clinical studies. For routine clinical use CARLO^®^ will have to be certified for each indication or for generic bone cutting.

With our report, we like to share our first results, enthusiasm and encourage researchers and clinicians to contribute to the field of robot‐guided and navigated laser osteotomies in the field of hand, wrist and forearm surgery.

## AUTHOR CONTRIBUTIONS

Philipp Honigmann, Maximilian Hofer, Sibylle Hirsch, Marta Morawska and Enrico Coppo conducted the study. Philipp Honigmann wrote the manuscript, which has been revised by Maximilian Hofe, Sibylle Hirsch, Marta Morawska, Magdalena Müller‐Gerbl, Florian M. Thieringer and Enrico Coppo. Magdalena Müller‐Gerbl provided the experimental surrounding. Marta Morawska and Sibylle Hirsch were responsible for the technical execution, Maximilian Hofe conducted a master thesis as basis for this study and was involved in the planning and execution of this study.

## CONFLICT OF INTEREST

The authors declare no potential conflicts of interest with respect to the research, authorship, and/or publication of this article.

## Data Availability

Research data are not shared.
